# Peptide Drug: Design and Clinical Applications

**DOI:** 10.1002/mco2.70287

**Published:** 2025-07-25

**Authors:** Yaqi Han, YunKui Zhang, Han Li, Zhongliang Ma, Yanmao Wang

**Affiliations:** ^1^ Lab for Noncoding RNA & Cancer School of Life Sciences Shanghai University Shanghai China; ^2^ Department of Anesthesiology Fudan University Shanghai Cancer Center Shanghai China; ^3^ Shanghai Sixth People's Hospital Affiliated to Shanghai Jiao Tong University School of Medicine Shanghai China

**Keywords:** circRNA‐encoded polypeptides, delivery strategy, immune performance, metabolic reprogramming, peptide drug

## Abstract

Peptide drugs possess superior biocompatibility and excellent specificity, making them a reliable choice in clinical treatment. They exert critical roles in disease‐associated metabolic reprogramming and immune modulation by activating cell signaling pathways, regulating metabolic processes, and immune cell functions. Notably, circular RNAs (circRNAs) have been shown to encode functional polypeptides. This finding offers new avenues for peptide drugs development. However, a summary of circRNA‐encoded polypeptides as peptide drug applications is relatively lacking. Therefore, we summarize the latest scientific advances in peptide drugs in the realm of diseases, with the focus on circRNA‐encoded polypeptides. We first delve into the functional mechanisms of peptide drugs within disease‐associated metabolic reprogramming and immune response. Subsequently, we provide an overview of the delivery and modification strategies of peptide drugs. Additionally, we summarize the encoding mechanisms of circRNAs and review the drug‐like applications of the polypeptides. We also highlight the potential challenges in the future development of circRNA‐based polypeptide drugs. In summary, we offer a systematic review of the research progress on circRNA‐encoded polypeptides, with the aim of providing novel perspective and ideas for the design and development of peptide drugs.

## Graphical abstract

The diagram illustrates the role of poly peptides in various biological processes. It highlights their involvement in glucose, lipid, and amino acid metabolism, as well as in antitumor and antiviral immunity. Additionally, poly peptides are linked to disease diagnosis and prognosis, emphasizing their significance in healthcare.

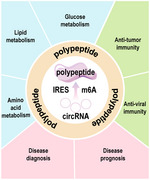

## 1 Introduction

Over a century ago, Frederick Banting's team successfully isolated insulin from the pancreas of livestock and confirmed its remarkable therapeutic efficacy in patients with type I diabetes [1, 2]. This event marked the birth of the first peptide‐based therapeutic drug. Subsequently, due to the development of synthetic technology, purification method, recombinant technology, and the creation of other peptide drugs [3, 4, 5], the field of peptide drugs has experienced substantial growth. To date, more than 100 peptide drugs have been approved for marketing worldwide [6, 7].

Peptide drugs are short‐chain polymers consisting of amino acid monomers, and the molecular weight of peptide drugs is between that of small molecule compounds and proteins [8, 9, 10]. Studies have found that these peptide drugs can positively mimic or interfere with physiological signals within the body [11, 12, 13, 14]. Additionally, amino acids are the metabolic end‐products of peptide drugs and generally cause no side effects on the body, suggesting that peptide drugs possess outstanding biocompatibility [15, 16]. Peptide drugs also exhibit superior efficacy and specificity in clinical treatment [17]. Therefore, peptide drugs have been extensively applied in the field of biomedicine, covering the clinical management of tumors, cardiovascular diseases, metabolic disorders, acromegaly, as well as other diseases [18, 19, 20, 21, 22, 23]. Specifically, in cancer therapy, peptide drugs have been shown to specifically recognize and bind to receptors on the surface of tumor cells or activate the antitumor signaling pathways, thereby exerting antitumor capability [18, 19]. In the treatment of metabolic diseases, glucagon‐like peptide‐1 (GLP‐1) analogs regulate insulin secretion and improved the body's glucose metabolism [24]. For acromegaly, octreotide, a somatostatin analog, inhibits the secretion of growth hormone and effectively controls the progression of disease [22, 23]. In the treatment of cardiovascular diseases, Evogliptin, a DPP‐4 inhibitor, has been shown to prevent diabetic cardiomyopathy [20].

Peptide drugs are also involved in abnormal energy metabolism in disease‐associated metabolic reprogramming [25, 26]. The reprogrammed metabolic processes embrace the reorganization of the utilization of diverse metabolic substrates [27, 28, 29]. The initiation and persistence of abnormal metabolism can alter cellular fate and also disturb traditional signal transduction pathways [30], thereby creating an environment conducive to disease onset and progression [31, 32]. In addition, it has been discovered that peptide drugs also mediate immune performance and disease resistance in individuals, offering a promising peptide‐based treatment strategy for immunotherapy field [33, 34].For instance, the Pal‐DMPOP chimeric peptide was found to highly improve immune performance mediated by macrophages [35]. However, the limited delivery efficiency and stability in vivo of peptide drugs remain major obstacles to further clinical application [36].

Recently, researchers have identified a novel class of polypeptides encoded by circular RNAs (circRNAs). A wealth of studies has shown that these unique polypeptides play important roles in promoting or inhibiting the occurrence and course of various diseases [37, 38, 39, 40]. This discovery suggests the potential of circRNA‐encoded polypeptides as novel therapeutic agents or drug targets. However, there is a lack of systematic reviews of circRNA‐encoded polypeptides in drug development and clinical applications.

Therefore, this review provides a comprehensive overview of the potential of peptide drugs in metabolic reprogramming and immune regulation, focusing on the unique functions and therapeutic value of circRNA‐encoded polypeptides. We also analyze the feasibility of utilizing these polypeptides as novel peptide drugs or drug targets. Furthermore, we summarize the current strategies for delivering peptide‐based drugs. In summary, this review seeks to offer a new perspective for the future advances of peptide drugs and promote the wide application of peptide drugs in clinical therapy.

## 2 Peptide Drugs and Metabolism Reprogramming in Diseases

Metabolism reprogramming is an intrinsic feature of various diseases, serving to fulfill the altered particular demands of the pathophysiological state [41, 42, 43, 44]. It has been found that peptide drugs exhibit therapeutic effects on diseases by regulating the activity of specific metabolic enzymes or metabolic processes. For instance, GLP‐1 is a peptide hormone that effectively regulates individual blood glucose levels. Currently, GLP‐1 receptor agonists, such as liraglutide and semaglutide, effectively mimic the effects of GLP‐1 to regulate energy metabolism and exhibit therapeutic role in the treatment of diabetes and obesity [24, 45].

### 2.1 Peptide Drugs and Diseases‐Associated Glucose Metabolism

The reprogrammed glucose metabolism stands as the most prevalent metabolic reprogramming process [46, 47]. Cai and colleagues [48] identified lactate involved in modulating mitochondrial oxidative phosphorylation and hindering glucose fermentation. For instance, tumor cells exhibit a powerful preference for the glycolytic pathway, a phenomenon known as the “Warburg effect” [49, 50]. Bartman and colleagues [51] found that the tricarboxylic acid (TCA) cycle flux in solid tumors of mice was lower than in almost all healthy tissues, which led to an increase in glycolysis flux (Warburg effect).

Tang et al. [26] found that semaglutide exhibits favorable cardioprotective effects. Semaglutide treatment significantly improves mitochondrial respiratory dysfunction in mice with heart failure [26]. The hearts of healthy individuals rely primarily on fatty acid oxidation for energy. However, cardiac metabolism in patients is disrupted, with reduced lipid oxidative capacity and TCA cycle flux, alongside increased glycolytic capacity [52, 53]. In their research, they found a strong correlation between semaglutide and the phosphatidylinositol 3‐kinase/protein kinase B (PI3K/AKT) signaling pathway. RNA‐sequencing data and western blot (WB) results showed that the expression and translocation of nuclear receptor subfamily 4 group A member 1 (NR4a1) to mitochondria were downregulated by semaglutide. This restrained the binding of NR4a1 to cAMP response element‐binding protein 5 (Creb5), thereby improving glucose and lipid metabolism disorders in the heart. In addition, they found that semaglutide promotes pyruvate entry into mitochondria and restores energy supply.

Doxorubicin is a potent chemotherapeutic agent for cancer but is associated with cardiotoxicity [54, 55]. Li et al. [56] found that semaglutide improved mitochondrial energy metabolism activity by reducing the expression of BNIP3 in mitochondria. RNA sequencing and qRT‐PCR shown that BNIP3 was highly upregulated, implying that it may be a candidate molecule responsible for doxorubicin cardiotoxicity. Subsequently, they injected adeno‐associated virus serotype 9 carrying BCL2/adenovirus E1B 19kDa interacting protein 3 (BNIP3) expression into the tail vein of C57/BL6J mice. The relevant results showed that the overexpression of BNIP3 abrogated the improvement of mitochondrial function induced by semaglutide. IRW (Ile–Arg–Trp) is a bioactive peptide and possesses promising therapeutic efficacy [57]. Son and Wu [58] found that tumor necrosis factor‐alpha (TNF‐α) treatment increased the phosphorylation levels of p38 and c‐Jun N‐terminal kinase (JNK) 1/2 proteins and affected the glucose uptake capacity of cells. However, the phosphorylation of p38 and JNK1/2 was inhibited by IRW, and insulin sensitivity was restored [58]. In addition, di‐peptide Trp–His(WH) was found to enhances glucose uptake ability by activating AMPK pathway [59].

### 2.2 Peptide Drugs and Diseases‐Associated Lipid Metabolism

Lipids cover an array of lipid molecules, containing fatty acids, triglycerides (TGs), phospholipids, and cholesterol [28, 60]. Remodeling of lipid metabolism regulates the composition and distribution of intracellular lipids, thereby influencing the cellular physiological status and functional performance [61, 62, 63].

Tooyserkani et al [64]. found that glucose‐dependent insulinotropic peptide highly inhibits the de novo synthesis of fatty acids. Moreover, this peptide drug regulated individual fatty acid storage capacity by increasing the production of glycerol required for TG production [64].

Liraglutide was found to restrain the increase of serum TG, total cholesterol, and ectopic lipid droplet deposition in renal tubules caused by diabetic nephropathy. This effect was mediated by downregulation of sterol regulatory element‐binding protein 1 (SREBP‐1) and fatty acid synthase, along with upregulated the expression of adipose TG lipase (ATGL) and hormone‐sensitive lipase [65].

Nesfatin‐1 and nesfatin‐1‐like peptides caused a reduction in intracellular lipid content by impairing fatty acid synthesis and increasing fatty acid oxidation. In addition, the inhibitor of AMP‐activated protein kinase (AMPK) compound C blocked the inhibitory effect of the drug on lipid content, indicating that its action depends on the AMPK pathway [66].

### 2.3 Peptide Drugs and Diseases‐Associated Amino Acid Metabolism

The disorder of amino acid metabolism is closely related to neurological disorders, metabolic diseases, and various tumors [67, 68, 69, 70]. Studies have also shown that abnormal amino acid metabolism led to immune damage [71, 72]. Notably, it has been observed that cancer cells exhibit an increased in the uptake and consumption of glutamine to meet the elevated need for biosynthesis and energy. Glutamine is a critical spring of carbon and nitrogen in cellular metabolism. A classic example of amino acid metabolic reprogramming is the phenomenon of glutamine addiction [73, 74].

Telaglenstat (CB‐839) is an allosteric inhibitor of glutaminase (GLS) that limits the metabolic transition of glutamine to glutamate by inhibiting GLS activity. Cai et al. [75] have found that CB‐839 regulated amino acid metabolism in acute myeloid leukemia cells. In addition, the combination of CB‐839 and 5‐azacytidine inhibited the growth of acute myeloid leukemia (AML) cells [75]. Furthermore, in a myeloproliferative tumor mouse model, Usart et al. [76] found that inhibition of glutamine metabolism with CB‐839 alleviated polycythemia.

968 is a pan‐GLS inhibitor. Wang et al. [77] demonstrated that the combination of 968 and a programmed death‐ligand 1 (PD‐L1) blocker enhanced the tumor immune response in ovarian cancer. 968 inhibited the proliferation of ovarian cancer cells and elevated the apoptosis rate of cancer cells. In addition, 968 enhanced the secretion of granzyme B by CD8+ T cells. The in vivo studies have shown prolonged survival benefit with 968 in combination with anti‐PD‐L1[77].

These findings suggest that peptide‐based drugs affect energy metabolism by regulating glucose uptake capacity or through related metabolic pathways.

## 3 Peptide Drugs in Modulating Immune Response Intensity

Immunopathology is an interdisciplinary field that studies the interactions between the immune system and disease processes. Its concern concentrates on the immune system's response mechanisms under pathological stimuli.

During infectious diseases, the immune system contributes powerful defense against invading pathogens [78]. By contrast, abnormal immune activities in autoimmune diseases mistakenly target the organism's tissues and cells [79]. In addition, tumor immunity controls the biological behavior of tumors by modulating the immune system's responses [80, 81]. Interestingly, a lot of studies have demonstrated that peptide drugs participate in modulating the activity of immune cells and inflammatory responses.

Liu et al. [82] developed a small molecule peptide, CLP002 peptide, that specifically binds to PD‐L1. This targeted peptide bound to PD‐L1 with high affinity and blocked programmed cell death protein 1 (PD‐1)/PD‐L1 signaling. In addition, the targeted peptide upregulated the level of interferon‐γ and elevated the tissue infiltration of CD8+T cells, thus exerting excellent anticancer effect [81].

Using a phage display combinatorial peptide library, the researchers found Pep‐39 peptide targets PD‐L1. Pep‐39 peptide was found to inhibit the expression of PD‐L1. The coimmunoculture experiments showed that Pep‐39 promoted the proliferation of Jurkat cells. These results indicated that Pep‐39 possessed a blocking effect on PD‐1/PD‐L1 interaction [64].

Lipopolysaccharide (LPS) possess the capability to induce strong immune responses within cells and individuals. According to Novoselova's research [83], the lipopolysaccharide‐mediated inflammatory activation of RAW264.7 macrophages was significantly improved in the presence of thymosin‐1α. It was found that thymosin‐1α could downregulate nuclesr factor kappa B (NF‐κB) and stress‐activated protein kinase (SAPK)/JNK signaling cascade expansion. Specifically, the addition of thymosin‐1α normalized the levels of interleukin‐1β and interleukin‐6. In addition, thymosin‐1α highly reduced the activity of p53 gene, resulting in the lower apoptosis rates of RAW264.7 macrophages. These findings suggest that thymosin‐1α acts as a vital regulator in mediating immune responses and suppressing inflammation [83].

## 4 Optimization Strategies for Peptide Drugs

The optimization strategy of peptide drugs aims to improve their delivery efficiency and stability. At present, researchers have developed a variety of alternative delivery systems to elevate delivery efficiency, including nanocarriers, liposomes, polymer micelles, and others [84, 85, 86]. Additionally, modification strategies cover the addition of unnatural amino acids and chemical modifications [4, 87, 88].

Nanodrug delivery systems (NDDS) are technologies that utilize nanomaterials to load peptide drugs with the aim to enhance drug efficacy by improving delivery efficiency [89]. Self‐assembling peptide is an important part of NDDS and drives molecular assembly into regular nanostructures with higher physicochemical stability [90]. Li et al. [91] used succinic acid to form a conjugate between the RGD (Arg–Gly–Asp) peptide and paclitaxel. Subsequently, experiments showed that this binding spontaneously formed nanofibers (P‐NFs) and exhibited great effective than paclitaxel alone in gastric cancer (GC) [91].

In the aqueous phase, lipid molecules spontaneously form double‐layer hollow vesicles through hydrophobicity interactions. This structure can not only wrap hydrophilic drugs inside the glomerulus, but also utilize lipophilicity to load lipophilic drugs between the lipid bilayer [92, 93]. For instance, Jiang et al. [94] used dendritic peptides to construct the dendritic liptide liposome delivery platform, showing outstanding efficacy of intramolecular mitochondrial targeted delivery.

Extracellular vesicles (EVs) are membrane‐encapsulated structures that serve as carriers for an assortment of biomolecules [95]. The transported cargoes include pathogenic entities, such as disease‐specific proteins and peptides, as well as various mRNAs and ncRNAs [96, 97]. EVs are categorized based on size into exosomes (<150 nm), microvesicles (100–1000 nm), and apoptotic bodies (>1000 nm) [98]. Under pathological settings, cells produce and release a substantial quantity of exosomes, with yields markedly exceeding that of normal cells [99].Moreover, the dimensions tend to exceed typical ranges [100].

In terms of the deliverability of EVs, the team of Jayasinghe et al. [101] used red blood cells‐derived EVs to develop a new strategy for targeted delivery of therapeutic polypeptide drugs for the treatment of leukemia. Pham et al. [102] combined EVs with peptide drugs and nanoantibodies achieved targeted delivery of EVs. Therefore, the combination of peptide drugs with EVs delivery systems is an innovative strategy that leverages the natural biocompatibility and targeting capabilities of EVs.

In addition to optimizing the properties of existing peptide drugs, the exploration of novel peptide drugs should be equally valued. The polypeptides encoded by circRNAs are novel natural polypeptide recently identified in vivo, and it is of great significance to carry out related research on these polypeptides.

## 5 CircRNA‐Encoded Polypeptides as Potential Candidates of Peptide Drugs Fields

The coding potential of circRNAs is a current research hotspot. In addition, circRNAs serve as central hubs in circRNA–miRNA–mRNA (messenger RNA) interplay networks [103, 104, 105]. They also govern transcription, function as protein sponges [106, 107, 108, 109, 110].

An increasing number of studies have revealed that circRNA‐encoded polypeptides possess independent biological functions. For instance, the polypeptide circKANSL1L‐511aa, translated by circKANSL1L, was found to interact with the AKT protein. This interaction suggested that circKANSL1L‐511aa was involved in muscle formation and muscle fiber composition [111]. BIRC6‐236aa, encoded by circBIRC6‐2, was linked to transmissible gastroenteritis virus (TGEV)‐induced mitochondrial dysfunction [112]. In addition, circPPP1R12A‐73aa (hsa_circ_0000423) [113, 114], CM‐284aa (hsa_circ_0069982) [115], and circFGFR1p (hsa_circ_0084007)[116] also exhibited unique function within different diseases. Furthermore, this coding capacity may facilitate cellular adaptation to certain environmental stresses. For example, research conducted by Pamudurti's team [117] has uncovered that starvation can regulate the translation of a circMbl isoform.

### 5.1 The Mechanism of CircRNA‐Encoded Polypeptides

The translation processes of circRNAs are typically mediated by internal ribosome entry sites (IRES) elements or modulated by modifications of N6‐methyladenosine (m6A) nucleotide sequences [116, 118]. In addition, a portion of circRNAs also perform translation through the rolling circle translation (RCT) mechanism or via the homologous untranslated regions (UTRs) of pre‐mRNAs [117, 119, 120]. These events result in the synthesis of functional polypeptides that may incorporate protein‐like domains [117, 121, 122, 123].

#### 5.1.1 IRES‐Mediated Synthesis of CircRNA‐Encoded Polypeptides

Initially, the coding potential of circRNAs was identified in artificial cyclization vectors that incorporated IRES [124]. This groundbreaking discovery has indeed laid a solid foundation for the subsequent scientific research in the field of circRNA‐encoding.

As a special sequence, IRES can directly engage with the ribosome or interact with other translation initiation factors to promote the translation of circRNAs [121, 125, 126]. IRES is usually located in the 5′UTR with a median length of 174 bp [127, 128, 129]. The IRES elements embedded within circRNAs act as a pivotal factor for translation. The IRES‐reliant internal ribosome entry system allows ribosomes to combine to circRNAs and perform the synthesis of functional polypeptides [116, 130].

Yang et al. [131] employed a dual‐luciferase vector system to ascertain the effective IRES activity of circ‐FBXW7, and the protein band of encoded product FBXW7‐185aa was detectable in both glioblastoma (GBM) cells and 293T cells. They also discovered that the malignant traits of GBM were enhanced by knockdown of FBXW7‐185aa. In parallel, the encoding of AKT3‐174aa was driven by IRES of hsa_circ_0017250 at the 118–333 nucleotide positions [132]. Song and colleagues [133] demonstrated that circZKSaa in HCC was driven by IRES 134–284 and 143–293 of circ‐ZKSCAN1 (hsa_circ_0001727).

Additionally, in GBM, the polypeptide circHEATR5B‐881aa was identified and shown to suppress cell proliferation within GBM cells [134]. Similarly, AXIN1‐295aa in GC was implicated in the regulation of cellular processes, controlling malignant behaviors of cancer cells [135]. In lung adenocarcinoma (LUAD), ASK1‐272aa was found to be associated with the suppressive effect on gefitinib resistance [136]. PDE5A‐500aa was identified as a specific polypeptide in esophageal squamous cell carcinoma (ESCC), suppressing the expansion and metastasis of ESCC cells [137]. PDHK1‐241aa was identified in clear cell renal cell carcinoma (ccRCC) and it promoted the proliferation, migratory behavior, and invasion within ccRCC cells [138]. Furthermore, HER2‐103aa emerged as a critical role in the tumorigenicity of triple negative breast cancer (TNBC) [139]. Circ‐EIF6 was found to encode the EIF6‐224aa polypeptide through an IRES element, which located 150 base pairs from the initiation codon. This polypeptide was shown to decrease the degradation of MYH9 by inhibiting the ubiquitin–proteasome pathway, which in turn led to the activation of the Wnt/β‐catenin pathway. This process promoted the proliferation and metastasis of TNBC [140]. Particularly, circEPS15 depended on its internal IRES to encode a 150aa polypeptide, and the translation process relied primarily on IRES2 rather than IRES1 [141]. They only identified the coding products of circEPS15 and did not conduct any follow‐up experimental studies. The context‐specific details are presented Table 1.

**TABLE 1:** IRES mechanism driven circRNA‐encoded polypeptides.

CircRNA	IRES positions	Polypeptide	Polypeptide function	Diseases	References
circ‐BIRC6‐2	1–174 590–763	BIRC6‐236aa	Acts as a decoy for components of mitochondrial permeability transition pore (mPTP), limiting TGEV‐induced cell death	TGEV	[112]
circ‐FBXW7	57–120	FBXW7‐185aa	Interferes the interaction of USP28 with FBXW7α, reducing the stability of c‐Myc	GBM	[131]
circ‐AKT3	118–333	AKT3‐174aa	Competitive interaction with phosphorylated PDK1, and negatively regulates PI3K/AKT signal strength	GBM	[132]
circ‐ZKSCAN1	134–284 143–293	circZKSaa	Promotes mTOR ubiquitination to inhibit PI3K/AKT/mTOR signal strength	HCC	[133]
circ‐HEATR5B	707–853	circHEATR5B‐881aa	Regulates M2 type pyruvate kinase (PKM2) activity and mediate aerobic glycolysis and proliferation	GBM	[134]
circ‐AXIN1	115–257 689–838	AXIN1‐295aa	Competitive binding to APC leads to β‐catenin release and nuclear translocation, transactivating the canonical Wnt pathway	GC	[135]
circ‐GSPT1	243–311 498–572	GSPT1‐238aa	Through the PI3K/AKT/mTOR signal pathway to regulate autophagy	GC	[142]
circ‐ASK1	588–701	ASK1‐272aa	Induced the apoptosis of cells and increases the sensitivity of gefitinib	LUAD	[136]
circ‐PDE5A	921–1094	PDE5A‐500aa	Interacts with PIK3IP1 and promotes USP14‐mediated deubiquitination, inhibiting the PI3K/AKT pathway	ESCC	[137]
circ‐PDHK1	112–261	PDHK1‐241aa	Inhibits AKT dephosphorylation and activates the AKT–mTOR signaling pathway	ccRCC	[138]
circ‐HER2	438–586	HER2‐103aa	Promotes homo/hetero dimerization of EGFR/HER3, sustains AKT phosphorylation	TNBC	[139]
circ‐EIF6	No information^a^	EIF6‐224aa	Activates the MYH9/Wnt/β‐catenin pathway and promotes cancer progression	TNBC	[140]
circ‐SHPRH	283–409	SHPRH‐146aa	Protects full‐length SHPRH from degradation by the ubiquitin proteasome	Glioma	[143, 144]
circ‐HGF	47 nucleotides	C‐HGF‐119aa	Direct interaction with the c‐MET receptor leads to autophosphorylation and activation of the receptor	GBM	[145]
circ_0036176	No information^a^	Myo9a‐208	Inhibits cell proliferation and cell cycle	Cardiac field	[146]
circTmeff1	1–174	TMEFF1‐339a	Reduce the myotube diameter and inhibit AKT/FOXO3A/mTOR signaling pathway	Muscle atrophy	[147]
circ0003692	74–469	FNDC3B‐267aa	Interacts with c‐Myc and promotes proteasome degradation of c‐Myc	GC	[148]
circFNDC3B	448–524	circFNDC3B‐218aa	Suppresses the epithelial–mesenchymal transition (EMT) progression by regulating the Snail/fructose‐1,6‐bisphosphatase 1 (FBP1) signaling axis	Colon cancer	[149]
circMRCKα	391–525	circMRCKα‐227aa	Promotes glucose uptake capacity, lactic acid production efficiency, and glycolysis levels within cancer cells	HCC	[150]
circINSIG1	207–292	circINSIG1‐121aa	Suppresses INSIG1/SREBP2 axis, triggering cholesterol biosynthesis and enhancing the proliferative and metastatic capabilities of cells	CRC	[41]
circMYBL2	278–549	p185	Disrupts the interaction between UCHL3 and the PHGDH, inhibiting the biosynthesis of serine and glycine	CRC	[151]
circYthdc2	250–337 338–424	Ythdc2‐170aa	Promotes ubiquitination degradation of STING protein and inhibit STING‐mediated antiviral response, regulating viral replication	Antiviral immune	[152]

Abbreviations: AKT, protein kinase B; APC, adenomatous polyposis coli; c‐MET, c‐mesenchymal‐epithelial transition factor; ccRCC, clear cell renal cell carcinoma; CRC, colorectal cancer; EGFR, epidermal growth factor receptor; ESCC, esophageal squamous cell carcinoma; FBP1, fructose‐1,6‐bisphosphatase 1; FOXO3A, Forkhead box protein O3A; GBM, glioblastoma multiforme; GC, gastric cancer; HCC, hepatocellular carcinoma; HER3, human epidermal growth factor receptor 3; INSIG1, insulin‐induced gene 1; LUAD, lung adenocarcinoma; mTOR, mammalian target of rapamycin; MYH9, myosin heavy chain 9; PDK1, phosphoinositide‐dependent kinase‐1; PHGDH, phosphoglycerate dehydrogenase; PI3K, phosphatidylinositol 3‐kinase; SREBP2, sterol regulatory element‐binding protein 2; STING, stimulator of interferon (IFN) genes; TGE, transmissible gastroenteritis virus; TNBC, triple‐negative breast cancer; UCHL3, ubiquitin carboxyl‐terminal hydrolase L3; USP, ubiquitin‐specific protease; Wnt, Wnt signaling pathway.

^a^Within the scope of the particular essay, no specific modification sites were investigated.

**TABLE 2:** m6A mechanism driven circRNA‐encoded polypeptides.

CircRNA	m6A positions	Polypeptide	Polypeptide function	Diseases	References
circMAP3K4	A787 A862	circMAP3K4‐455aa	Reduce the cleavage and nuclear translocation of mitochondrial apoptosis‐inducing factor, thereby preventing cisplatin‐induced apoptosis in HCC cells and fulfilling as carcinogenic	HCC	[37]
circ‐β‐TrCP	No site information^a^	β‐TrCP‐343aa	Interacts with NRF2 to block its ubiquitination and degradation, forming a positive feedback loop with NRF2 that contraposes eIF3j, thereby activating trastuzumab resistance	BC	[153, 154]
circ‐ZNF609	No site information^a^	Unnamed polypeptide	Enhances the process of myoblast differentiation in mouse and human	Myogenesis	[155, 156]
circMET	No site information^a^	MET404	Interacts with the β subunit of MET to form a constitutionally activated MET receptor, activating AKT/ERK	GBM	[157]
circYthdc2	A91 A132	Ythdc2‐170aa	Promotes ubiquitination degradation of STING protein and inhibit STING‐mediated antiviral response, regulating viral replication	Antiviral immune of fish	[152]
circ‐YAP	GGACA (GGCCA)	YAP‐220aa	Competitive binding to LATS1 leads to YAP dephosphorylation and nuclear translocation	CRC	[158]
circMIB2	UGACA (UGCCA)	MIB2‐134aa	Targets to TRAF6 protein and promotes TRAF6‐mediated innate immune response	Antiviral immune of fish	[123]

^a^Within the scope of the relevant paper, no specific modification sites were investigated. Instead, the study referred to the broader aspects of the m6A methylation machinery, covering the m6A writers (METTL3, METTL14) and m6A readers (YTHDF1‐3, YTHDC1‐2). The former is responsible for the methylation event, while the latter dedicates to the identification reading of methylation information.

Abbreviations: BC, Breast cancer; eIF3j, eukaryotic translation initiation factor 3 subunit J; ERK, extracellular signal‐regulated kinase; LATS1, large tumor suppressor kinase 1; METTL, methyltransferase‐like; NRF2, nuclear factor erythroid 2‐related factor 2; TRAF6, TNF receptor‐associated factor 6; YAP, Yes‐associated protein; YTHDF, YTH domain family.

#### 5.1.2 m6A‐Mediated Synthesis of CircRNA‐Encoded Polypeptides

The modification of m6A is one of the most shared RNA modifications in eukaryotes and is ubiquitous in mRNAs [159, 160]. The modification pattern of m6A base is a reversible dynamic epigenetic marker, controlled by specific “writers,” “erasers,” and “readers” [161, 162]. The “writers” of m6A modification mainly include methyltransferase complexes, such as methyltransferase like 3 (METTL3), METTL14, and WTAP [163, 164]. For instance, METTL3 was found to bind to endogenous polyribosomes, thereby facilitating the translation process [165]. These complexes catalyze the formation of m6A modifications on adenosine (A) in mRNA. FTO and ALKB‐honolog 5 (ALKBH5) act as demethylases, responsible for removing the methyl groups (‐CH3) from the already existing m6A‐modified bases, achieving demethylation [166, 167, 168]. The “readers” of m6A modification recognize specific adenosine‐modified, thereby activating downstream molecular regulatory pathways [169, 170, 171, 172].

This m6A modification pattern controls almost all biological reactions and normal molecular functions within cells by influencing RNA splicing generation, physiological stability, translation behavior, and degradation of RNA [173, 174, 175, 176, 177, 178]. Moreover, abnormal m6A modification activity can potentially induce a series of diseases such as various tumors, cardiovascular disease, leukemia, and immune inflammation [161, 179, 180, 181, 182, 183, 184]. An increasing amount of experimental evidence suggests that m6A methylation modification acts a crucial role in a wide range of physiological and pathological biological processes [185, 186, 187]. In addition, Wang et al. [188] reported that m6A is the most abundant base modification in RNA and can effectively promote the translation of human circRNAs, although a very limited number of circRNAs are capable of being translated in such a manner. This special modification pattern alters the stability and structure of circRNA molecules, serves as an alternative mechanism for regulating translation [169, 189, 190, 191]. Specifically, m6A functions akin to an IRES, assisting the binding of circRNAs to ribosomes and pushing the encoding of circRNAs [159, 188, 192].

For instance, the m6A modification sites on the circMAP3K4 sequence were identified by Duan and colleagues [37]. The specific sites within the circMAP3K4 were recognized by the RNA‐binding protein (RBP) insulin‐like growth factor 2 mRNA‐binding protein1 (IGF2BP1). This RBP facilitated the translation of circMAP3K4 into a specific polypeptide, circMAP3K4‐455aa. In the initial phase of the research, they discovered that neither of the two IRES regions of circMAP3K4, spanning 83–210 and 1166–1302, were capable of initiating the translation action. Building upon these findings, further investigation identified that certain mutations within the circMAP3K4 sequence had significant effects on the expression levels of the circMAP3K4‐455aa polypeptide. Notably, both the single adenine mutation at position 862 (A862C) and the double adenine mutations at positions 862 and 787 (A862C and A787C) caused a decrease in the abundance of expression of circMAP3K4‐455aa. Moreover, these mutations also impacted the interaction of IGF2BP1 with circMAP3K4. Essentially, IGF2BP1 acts as the “reader” for m6A, recognizing and binding to the GG(m6A)C sequences located on circMAP3K4. These findings demonstrated that the translation of circMAP3K4 was mediated by the m6A mechanism, highlighting the importance of m6A modification in the regulation of circMAP3K4's translation. Additionally, this polypeptide interacted with the N‐terminus of the apoptosis‐inducing factor (AIF) protein, thereby attenuating the apoptotic response mediated by AIF in HCC cells [37]. This study partially elucidated the intrinsic mechanisms associated with the resistance of HCC to cisplatin treatment. The study also found that circMAP3K4 and the polypeptide it encoded could be a potential target for HCC therapy, especially for those HCC patients who have developed resistance to chemotherapy.

Wang and colleagues discovered that β‐TrCP‐343aa, translated by circ‐β‐TrCP, endowed trastuzumab resistance to tumors in breast cancer (BC) cells [153]. Eukaryotic initiation factor 3 (eIF3) is the initiator of the largest complex form of mediated translation, a multisubunit complex that includes eIF3a to eIF3m [193, 194, 195]. RNAi screening revealed that the knockdown of eIF3 only strongly interfered with the expression of β‐TrCP‐343aa [153]. Specifically, when eIF3a or eIF3b were knocked down using siRNA, the levels of β‐TrCP‐343aa were significantly decreases. However, the results of knocking down eIF3j were reversed, suggesting that eIF3j was negative correlation with β‐TrCP‐343aa. These results suggested that eIF3j may inhibit the translation of circ‐β‐TrCP by hampering the complex formation of eIF3a, eIF3b, and circRNA. In other words, eIF3j functioned as a translation repressor of circRNA. Choe et al. [165] found that the subunit of eIF3 directly interacted with METTL3, suggesting a correlation between eIF3 and m6A modification. Molecular mechanism revealed that the translational inhibition resided in the m6A modification within circ‐β‐TrCP. The m6A modification of circ‐β‐TrCP was considered to be a signal that favors the binding of eIF3j to the circRNA. Once bound, eIF3j caused the dissociation of other eIF3 subunits, ultimately culminating in a clear reduction in the translation efficiency of circ‐β‐TrCP. Similarly, Song et al. [154] found that eIF3j restrained the translational effectiveness of translatable circRNA circSfl. These findings implied a strong correlation between the encoding capability of β‐TrCP‐343aa and the m6A modification mechanism [153].

Legnini and colleagues [155] identified that circ‐ZNF609 (hsa_circ_0000615) is heavily loaded on polyribosomes, suggesting that this circRNA has the potential to be translated into polypeptides. To verify the coding capacity of circ‐ZNF609, Legnini and colleagues utilized an optimized circRNA expression vector established by Kramer and colleagues [155, 196]. Based on this expression vector, Legnini and colleagues successfully demonstrated the translation capability of circ‐ZNF609 in vitro. Furthermore, they used the CRISPR/Cas9 gene‐editing system in mouse embryonic stem cells to insert a 3×Flag coding sequence into a specific region of the endogenous ZNF609 gene, namely, the region where circ‐ZNF609 is located, thereby producing the specifical polypeptide encoded by circ‐ZNF609 with the 3×Flag tag. This confirmed that the specifical polypeptide was indeed encoded by circ‐ZNF609 and also indirectly verified the endogenous expression of this polypeptide. Functionally, the polypeptide encoded by circ‐ZNF609 was found to promote the differentiation process of myoblasts in mice and humans in vitro [155]. In addition, Di Timoteo et al. [156] demonstrated that the knockdown of METTL3 highly and specifically reduced the biogenesis of circ‐ZNF609 and the translation of circ‐ZNF609. However, the knockdown of YTHDC3 restrained the translation of circ‐ZNF609, instead of YTHDC1/2 [156]. Their research has enhanced our understanding of the m6A methylation mechanism that driven the translation of circ‐ZNF609. Huang et al. [197] have established a database of circRNAs called TransCric, which includes information on m6A modifications within circRNAs. Zhong and colleagues [157] discovered that a MET variant (MET404) consisting of 404aa was encoded by circMET, which is promoted by the m6A reader YTHDF2.

It is noteworthy that the research by Zheng et al. was the first to discover in lower vertebrates such as miiuy croaker (*Miichthys miiuy*) that circYthdc2 encoded the 170aa polypeptide, Ythdc2‐170aa [152]. This translation process was achieved through two translation strategies: IRES elements and m6A modification. The encoded polypeptide was involved in the antiviral immune process of fish. Researchers discovered that the IRES elements spanning regions 250–337 and 338–424 were indispensable for the translation of circYthdc2. The activity of these two segments of IRES was confirmed by dual‐luciferase reporter assay in conjunction with green fluorescent protein. Ythdc2 is the host gene of circYthdc2 and an m6A‐binding protein which can obtain the m6A modification information [198]. Therefore, the researchers considered whether m6A was participated in the encoding process of circYthdc2. The experimental results showed that the introduction of mutations at two m6A modification sites in the UTR of circYthdc2 caused a sharp decrease in Ythdc2‐170aa level. Moreover, m6A modification associated methyltransferase and m6A‐binding proteins increased Ythdc2‐170aa expression. However, demethylases associated with m6A modification decreased Ythdc2‐170aa expression. These results suggested that m6A modification also contributed to the translation of circYthdc2. Moreover, this translation mode of circYthdc2 and its function were found to be highly conserved from lower‐vertebrate species to higher mammals, demonstrating the diversity of circRNA coding methods and the evolutionary conservation of their protein‐encoding capabilities [152].

In addition, YAP‐220aa has been identified as a novel polypeptide in liver metastatic CRC, which was encoded by a particular circ‐YAP through the m6A‐mediated translation mechanism. Studies have shown that the highly conserved m6A modification site “GGACA” on circ‐YAP was crucial for the generation of YAP‐220aa. The corresponding protein product could not be detected when the first adenosine (A) at this site was replaced with cytosine (C), confirming the importance of this m6A modification site for circRNAs. Furthermore, Zeng et al. [158] also found that YAP‐220aa competitively coupled to LATS1, causing the de‐phosphorylation and nuclear translocation of YAP. These events led to the activation of multiple metastasis‐associated genes. Additionally, the expression of this peptide was highly upregulated in metastatic CRC tissues and cell lines and was correlated with poor patient prognosis [158].

Zheng et al. [123] identified a novel, uncharacterized polypeptide encoded by circMIB2 in fish, which named as MIB2‐134aa. It was found that circMIB2 originated from the maternal gene E3 ubiquitin–protein ligase (MIB2), and prediction results showed that it has encoding capability. However, further research revealed that its sequence lacked IRES elements but contained an m6A modification site. This implied that circMIB2 might rely on m6A to perform encoding function. Based on these preliminary findings, their research team constructed a point mutation expression vector targeting the m6A modification site (Flag–circMIB2–m6A–mut‐P) in subsequent experiments. The sequence “UGACA” was mutated to “UGCCA.” The experimental results showed that the expression level of MIB2‐134aa from the mutated vector was significantly lower than that from the wild‐type vector, proving the “UGACA” modification site was indispensable for circMIB2 encoding. MIB2‐134aa and MIB2 contain the similar TNF receptor‐associated factor 6 (TRAF6)‐binding protein domains, and TRAF6 can mediate innate immune responses. Functionally, the roles of MIB2 and MIB2‐134aa are completely opposite. The former induced the ubiquitination and degradation of TRAF6, inhibiting the host's immune response and immune presentation, while the latter reversed the negative effects of the former on TRAF6, protecting the host from pathogenic bacterial invasion and colonization [123]. The details for the relevant points are presented in Table 2.

#### 5.1.3 Additional Strategies‐Mediated Synthesis of CircRNA‐Encoded Polypeptides

Besides the IRES element and m6A modification, there are also some other uncommon mechanisms that drive encoding. The pre‐requisite for circRNAs to perform the RCT mechanism is the presence of an infinite open reading frame (iORF) in the RNA sequences. This means that the iORF contains only the initiation codon and lacks a termination codon [119], allowing the same sequence information could be repeatedly read during translation [199]. This enables the production of polypeptides with an actual length larger than the theoretical length.

For instance, through circRNA sequencing on mouse testes, Zhang et al. [120] identified a conserved circRNA between mice and humans, circRsrc1. Subsequently, they used heavy isotope‐labeled peptide technology confirmed the endogenous expression of Rsrc1‐161aa. It is worth noting that the actual detected length of Rsrc1‐161aa was 161 amino acids, which exceeded the theoretical length encoded by circRsrc1. These findings suggested that Rsrc1‐161aa should be encoded by the RCT mechanism [120]. The molecular mechanism revealed that Rsrc1‐161aa directly interacted with the mitochondrial protein complement component 1 Q subcomponent‐binding protein (C1qbp) and enhanced its binding affinity with mitochondrial mRNA. This process mediated the assembly and translation of mitochondrial ribosomes during spermatogenesis, as well as mitochondrial energy metabolism. The experimental results of the functional deficiency experiment showed that the loss of Rsrc1‐161aa inhibited the proliferation of spermatogonia and induced G2/M arrest of the cell cycle. Ultimately, these processes damaged the quantity and vitality of the mice's sperm, suppressing the fertility of male mice. Another instance is rolling‐translated epidermal growth factor receptor (EGFR) (rtEGFR) [119]. This polypeptide is encoded by circ‐EGFR (has_circ_0080229) and circ‐EGFR contained an iORF. What is interesting is that Liu et al. detected “ladder‐shaped” rtEGFRs near 35, 40, 55, and 70 kDa. Furthermore, their research demonstrated that rtEGFRs with different lengths were different translation termination options. These finding verified that circ‐EGFR was encoded through RCT mechanism. Functionally, rtEGFR bound to and maintained the membrane localization of EGFR. This behavior weakened EGFR's endocytosis and subsequent degradation. In addition, the absence of rtEGFR in brain tumor‐initiating cells resulted in reduced tumorigenicity and inhibited the course of GBM.

In general, the encoding of circRNAs is an intricate and diverse process involving a variety of molecular processes. These encoded mechanisms expand our understanding of the functions and potential of circRNAs and provide new avenues directions for future research.

### 5.2 CircRNA‐Encoded Polypeptides and Metabolism Reprogramming in Diseases

In terms of polypeptides encoded by circRNAs, the reprogrammed metabolic activities may be achieved through interactions between polypeptide segments and metabolic enzymes, transporters, or key molecules of in metabolic signaling pathway [134].

#### 5.2.1 CircRNA‐Encoded Polypeptides and Diseases‐Associated Glucose Metabolism

Song and colleagues [134] found that circHEATR5B was expressed at weak levels in GBM tissues and cells, and it encoded HEATR5B‐881aa. Subsequently, they constructed overexpression and knockdown vectors for the polypeptide, and following experiments found that the upregulation of HEATR5B‐881aa significantly reduced lactate production and glucose consumption within the GBM cells. Furthermore, the upregulation of HEATR5B‐881aa also inhibited tumor cell proliferation. Conversely, the knockdown of HEATR5B‐881aa showed the completely opposite effects, promoting glycolysis in GBM. In addition, they found that the cytoplasm‐localized HEATR5B‐881aa has a high binding affinity with Jumonji C‐domain‐containing 5 (JMJD5). Moreover, JMJD5 induces PKM2 activation, thereby regulating glucose metabolism reprogramming [200, 201]. Mechanistically, zinc finger CCHC‐type and RNA binding motif 1 (ZCRB1) upregulated the levels of circHEATR5B. Through the IRES mechanism, circHEATR5B encoded the polypeptide. When HEATR5B‐881aa bound to JMJD5, this polypeptide induced specific phosphorylation at the S361 site of the JMJD5 protein. The stability of JMJD5 after phosphorylation was poor, and at this time, low levels of JMJD5 increased the kinase activity of PKM2. As a rate‐limiting enzyme, the activation of PKM2 significantly inhibited the glycolytic capacity of GBM and the proliferation of tumor cells.

Pan et al. [149] detected circFNDC3B‐218aa with a molecular weight about 25 kDa in colorectal cancer, and the expression of this peptide was downregulated. Mass spectrometry analysis results showed that the length of the specific polypeptide fragment of circFNDC3B‐218aa was 16 amino acids. Subsequently, they conducted in vitro and in vivo experiments with the polypeptide, and the results consistently showed that the overexpression of circFNDC3B‐218aa restricted the proliferation, invasion, and migration abilities of colorectal cancer cells. Moreover, this polypeptide reduced the incidence of liver metastasis in colon cancer cells. Further analysis revealed that circFNDC3B‐218aa was negatively correlated with the progression of EMT. From a mechanistic perspective, circFNDC3B‐218aa promoted the transition of metabolic activities from glycolysis to oxidative phosphorylation by inhibiting the Snail–FBP1 signaling axis, ultimately suppressing the progression of EMT. This process referred to the downregulation of Snail expression and the elevation of FBP1 levels, which together limited the invasive and metastatic capabilities of colon cancer cells [149].

Yu and colleagues [150] used the Transwell coculture of immune cells and tumor cells and found that after coculturing of tumor‐associated macrophages (TAMs) with HCC cells, the glucose consumption of HCC cells significantly increased. Moreover, the glycolytic capacity of HCC cells was markedly enhanced. Subsequently, they used circRNAs sequencing technology and discovered that the expression of circMRCKα (hsa_circ_0003659) was induced by TAMs, suggesting that high levels of circMRCKα might promote glycolysis and tumor progression in liver cancer cells. They then discovered that circMRCKα has encoding potential and the endogenous expression of circMRCKα‐227aa was detected. The biological function of circMRCKα‐227aa showed that the high expression of this polypeptide enhanced the malignant behavior of tumor cells and the levels of glycolysis, leading to the deterioration of HCC progression. Mechanistically, circMRCKα‐227aa colocalized with the deubiquitinating enzyme USP22 in the nucleus. This process promoted the removal of ubiquitin moieties from HIF‐1α, leading to an increase in HIF‐1α protein levels. The increase of HIF‐1α triggered the activation of glycolysis‐related genes, including hexoknise 2 (HK2), lactate dehydrogenase A (LDHA), and glucose transporter 1 (GLUT1). This study revealed that circMRCKα‐227aa and TAMs could be the combined targets for HCC treatment [150].

**FIGURE 1:**
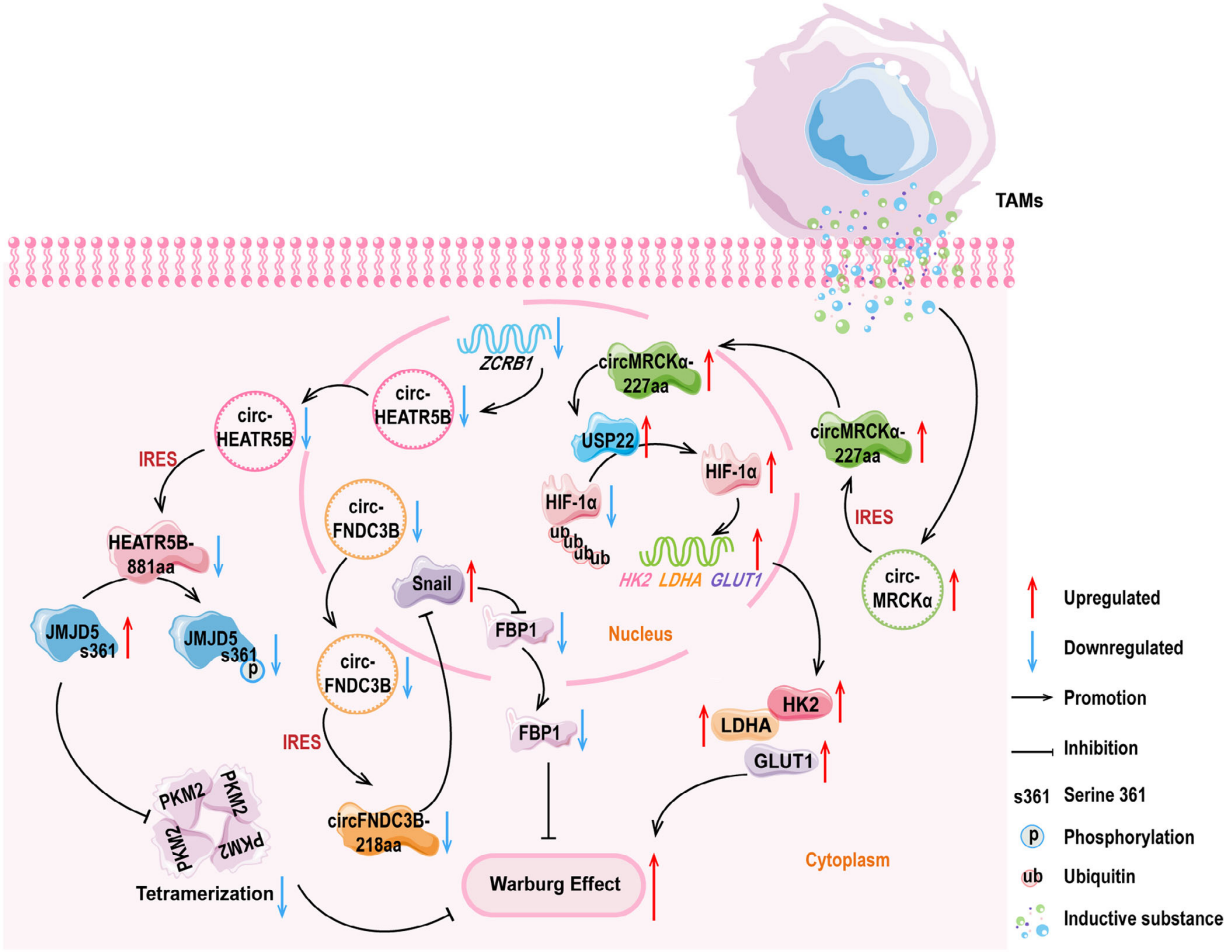
The schematic diagram depicts that polypeptides encoded by circRNAs are implicated in the regulation of the Warburg effect. CircRNAs are represented by ring structures, and the polygons of the same color connected to circRNAs by an arrow represents the circRNA‐encoded polypeptides. Other polygons represent proteins, and double helix‐like shapes represent DNA. Facilitation and inhibition are shown as arc‐shaped arrows, and upregulation and downregulation are shown as straight arrows. Irregular cells outside the phospholipid bilayer indicate tumor‐associated macrophages (TAMs). Blue and green dots indicate inducible substances released by TAMs. fructose‐1,6‐bisphosphatase 1; GLUT1, glucose transporter 1; HIF‐1α, hypoxia‐inducible factor 1‐alpha; HK2, hexokinase 2; IRES, internal ribosome entry site; JMJD5, Jumonji C domain‐containing protein 5; LDHA, lactate dehydrogenase A; PKM2, pyruvate kinase M2; Snail: Snail family transcriptional repressor; USP, ubiquitin‐specific protease; ZCRB1, zinc finger CCHC‐type and RNA binding motif 1.

The details for the aforesaid objects are presented in Figure 1.

#### 5.2.2 CircRNA‐Encoded Polypeptides and Diseases‐Associated Lipid Metabolism

The relationship between circRNAs encoded polypeptides and lipid metabolism is gradually being uncovered. The expression of p113, a polypeptide originating from the circRNA of the *CUX1* gene (hsa_circ_0005253) and reliant on an IRES element, was found to be highly elevated in neuroblastoma (NB) cells. The actual expression of p113 in cancers of NB, esophageal, lung, gastric, liver, cervical, prostate, and colorectal was detected by Yang and colleagues [202]. The findings indicated that p113 had a broad oncogenic effect. Further experiments revealed that p113 was primarily localized in the cell nucleus and the overexpression of p113 or its parent ecirCUX1 increased the intracellular fatty acid content, particularly palmitic acid, palmitoleic acid, oleic acid, stearic acid, and arachidonic acid. Essentially, it was demonstrated that p113 augmented the reprogramming of lipid metabolism in NB cells through the p113/zuotin‐related factor 1 (ZRF1)/bromodomain containing 4 (BRD4) trimeric complex [202]. Further experiments revealed that the artificially designed decoy protein ZIP‐12 could effectively bind to the p113 polypeptide, thereby limiting the activation capability of p113 during the process of lipid reprogramming.

The translation of circINSIG1 (hsa_circ_0133744) generated circINSIG1‐121aa that induced cholesterol biosynthesis and promoted CRC proliferation and metastasis. With respect to the translation mechanism, the study observed that circINSIG1‐121aa utilized the more ubiquitous IRES‐driven mechanism, specifically located within the 207–292 region of circINSIG1. Specifically, it was found that circINSIG1‐121aa restrained the protein level of insulin‐induced gene 1 (INSIG1) by elevating the ubiquitination levels at the 156/158 sites of lysine (K156/K158) within INSIG1, rather than through phosphorylation. Essentially, the study discovered that circINSIG1‐121aa acts as a decoy, attracting the CUL5–ASB6 complex and inducing the ubiquitination of INSIG1. This process has been shown to trigger cholesterol biosynthesis and improve the proliferation and metastasis ability of CRC cells. Additional experiments showed that the increase of circINSIG1 elevated the generation of SREBP2, in charge of cholesterol regulation. Conversely, it was found that overexpression of INSIG1 restrained SREBP2, demonstrating that the function of INSIG1 was opposite [41].

**FIGURE 2:**
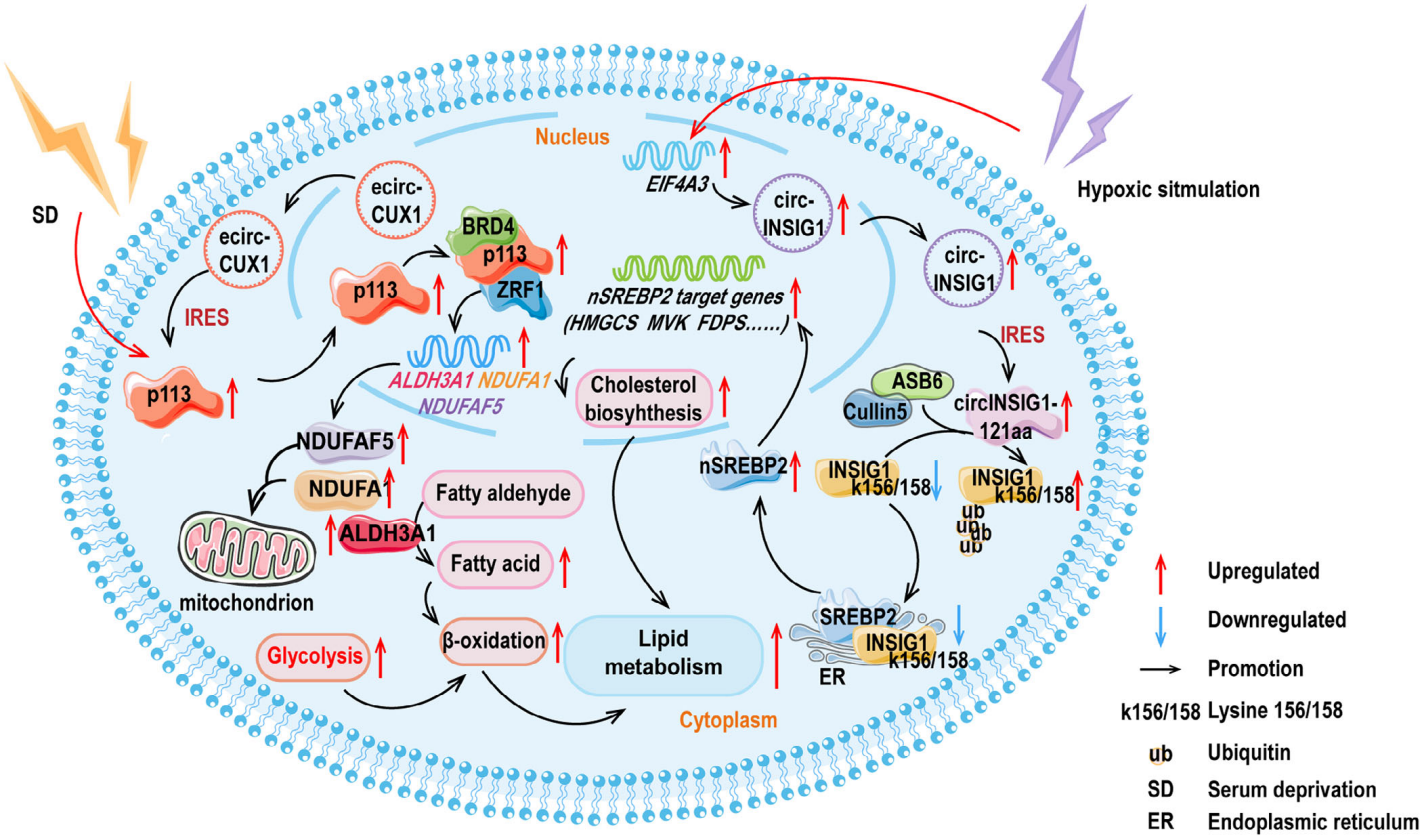
The schematic diagram illustrates that polypeptides encoded by circRNAs are implicated in the regulation of the metabolism of lipid. CircRNAs are represented by ring structures, and the polygons of the same color connected to circRNAs by an arrow represents the circRNA‐encoded polypeptides. Other polygons represent proteins, and double helix‐like shapes represent DNA. Facilitation is shown as an arc‐shaped arrow, and upregulation and downregulation are shown as straight arrows. The double‐layer membrane structure in the cytoplasm indicates the mitochondria, and the single‐layer reticular structure indicates the endoplasmic reticulum (ER). Lightning‐like shapes outside the phospholipid bilayer indicate exogenous stimuli. ALDH3A1, aldehyde dehydrogenase family 3 member A1; ASB6, ankyrin repeat and SOCS box protein 6; BRD4, bromodomain containing 4; EIG4A3, elongation initiation factor 4A3; FDPS, farnesyl diphosphate synthase; HMGCS, hydroxymethylglutaryl‐CoA synthase; INSIG1, insulin induced gene 1; MVK, mevalonate kinase; NDUFA1, NADH:ubiquinone oxidoreductase subunit A1; NDUFAF5, NADH:ubiquinone oxidoreductase complex assembly factor 5; nSREBP2, nuclear sterol regulatory element‐binding protein 2; SREBP2, sterol regulatory element‐binding protein 2; ZRF1, zuotin‐related factor 1

The details for the aforementioned points are rendered in Figure 2.

#### 5.2.3 CircRNA‐Encoded Polypeptides and Diseases‐Associated Amino Acid Metabolism

In terms of the polypeptides encoded by circRNAs, Zhao et al. discovered that circMYBL2 (hsa_circ_0006332) contained an IRES element located in its nucleotide sequence from positions 278 to 549. This IRES enabled the expression of p185. Additional studies have shown that p185 was poorly expressed in CRC cells. Overexpression of this 185‐amino‐acid polypeptide obviously restrained the proliferation of CRC cells. Furthermore, in vivo experiments have observed that p185 could significantly reduce the pulmonary metastasis of CRC, decreasing the amount and weight of pulmonary nodules. From the mechanistic standpoint, p185 was discovered to target the C1 domain of the deubiquitinating enzyme UCHL3, not only disrupting the interaction between UCHL3 and the substrate‐binding domain 2 (SBD2) of phosphoglycerate dehydrogenase (PHGDH) but also inhibiting the subsequent de‐ubiquitination and stabilizing effect of UCHL3 on PHGDH, leading to the maintenance of ubiquitination and degradation of PHGDH. Lysine residues at positions 146, 289, and 310 of PHGDH (K146/K289/K310) were identifies as the primary sites of action for UCHL3, with K310 being particularly crucial. Considering that PHGDH is the rate‐limiting enzyme for serine biogenesis, researchers confirmed using carbon‐13 (C^13^) labeled glucose that UCHL3 could increase the incorporation of C^13^ into newly synthesized serine and glycine by increasing PHGDH levels, implying that UCHL3 promoted the biosynthesis of serine and glycine. In conclusion, the polypeptides p185 encoded by circMYBL2 relied on the UCHL3/PHGDH pathway to control the biosynthesis of serine and glycine and acted as a tumor suppressor [151].

**FIGURE 3:**
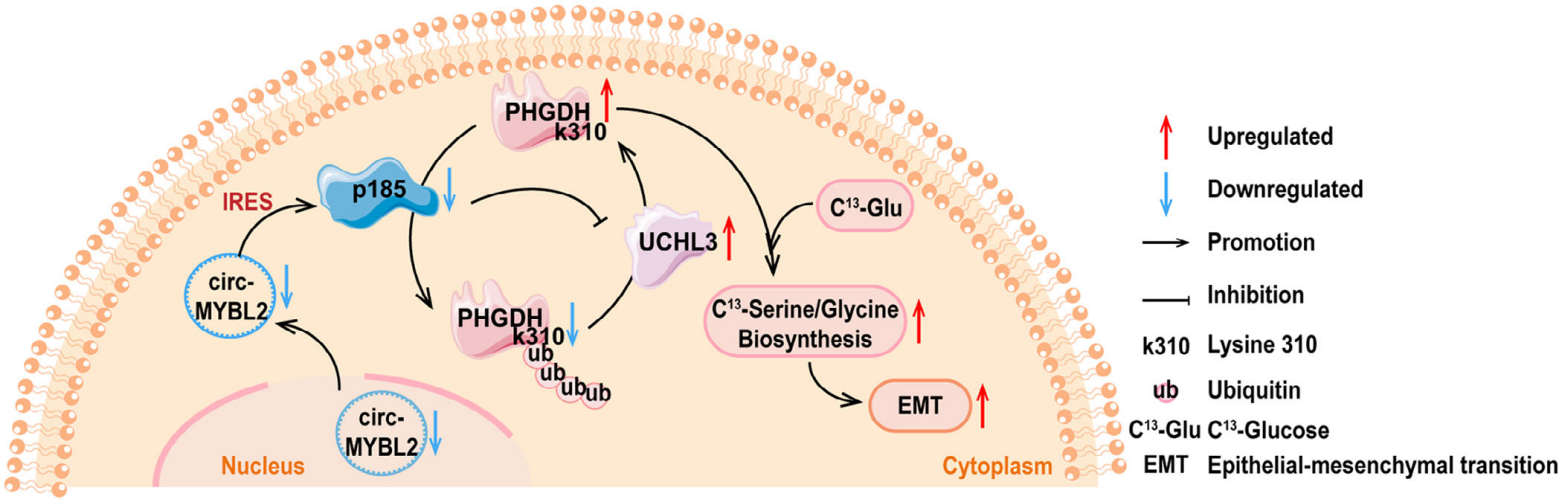
The schematic diagram illustrates that polypeptides encoded by circRNAs are implicated in the regulation of the biosynthesis of amino acid. CircRNA are represented by ring structures, and the polygon of the same color connected to circRNA by an arrow represents the circRNA‐encoded polypeptide. Other polygons represent proteins. Facilitation and inhibition are shown as arc‐shaped arrows, and upregulation and downregulation are shown as straight arrows. PHGDH, phosphoglycerate dehydrogenase; UCHL3, ubiquitin c‐terminal hydrolase L3.

The details for the above‐described points are presented in Figure 3.

#### 5.2.4 CircRNAs‐Encoded Polypeptides in Modulating Immune Response Intensity

Notably, Zhang et al. [118] unveiled that circKEAP1 activated antitumor immune response through retinoic acid‐inducible gene I (RIG‐I) and found that this circRNA possessed encoded capability. Therefore, there exists a plausible conjecture that specific polypeptides translated by circRNAs exhibited a profound impact on the functions and behaviors of immune cells by recognizing specific receptors. By controlling the immune activation, proliferation, and differentiation, this polypeptide modulated the intensity and direction of immune responses and acted as a key role in tumor course and immune evasion.

The remarkable finding is Song's research team made a valid discovery: a BC‐specific circFAM53B and its encoded polypeptide, circFAM53B‐219aa, could effectively triggered specific CD4+ and CD8+ T cells and facilitated a tumor immune response [38]. During this process, the polypeptide exhibited a strong binding affinity for major histocompatibility complex (MHC) class I and class II molecules, offering an effective route for the presentation of tumor antigens. In in vivo experiments, vaccination with this polypeptide highly enhanced the tissue infiltration of cytotoxic T cells specific to tumor antigen. This immune activation not only highly increased the immunological activity within the tumor microenvironment but also effectively controlled tumor growth, offering a novel immunological strategy for cancer treatment [38]. Phase II data from Koga et al. [203] showed that the combination of an herbal active ingredient and the personalized peptide vaccine highly enhanced the immune response in patients with castration‐resistant prostate cancer (CRPC). A Phase III clinical study by Noguchi et al. [204] demonstrated survival benefits in patients with metastatic CRPC during treatment with the personalized peptide vaccine. These findings exhibit at the promise of personalized vaccines and cell therapy products. More importantly, the implementation of the polypeptide strategy offered the possibility of personalized immunotherapy. The precise design of polypeptides enables them to bind with the patient's MHC molecules, pushing a personalized treatment concept for each patient, maximizing therapeutic efficacy, and minimizing unnecessary side effects [38].

Zheng et al. [123] discovered that circMIB2 effectively cleared and prevented pathogenic infections by encoding MIB2‐134aa. They constructed a series of vectors, including circMIB2, circMIB2–ATG–mut, and the expression vectors of immune signaling molecules covering stimulator of interferon genes (STING), MAVS, TIR‐domain containing adaptor‐inducing (TRIF), and TRAF6. Moreover, the gene reporter vectors containing interferon regulatory factor 3 (IRF3), interferon‐stimulayed response element (ISRE), and NF‐κB, respectively. The experimental results showed that overexpression of circMIB2 highly upregulated the protein level of TRAF6. Additionally, the luciferase activity of the reporter gene significantly increased only when the circMIB2 vector was cotransfected with the TRAF6 expression vector. There was no such regulatory trend in circMIB2–ATG–mut group, indicating that MIB2‐134aa activated individual immune signals by upregulating TRAF6. Additionally, the proteasome inhibitor MG132 effectively blocked the degradation of TRAF6 by MIB2. Therefore, the effects of MIB2‐134aa and MIB2 on TRAF6 were thoroughly opposite. Mechanistically, MIB2‐134aa and MIB2 contained similar TRAF6 binding domains, and MIB2‐134aa competitively bound to TRAF6, thereby exaggerating the antiviral immune response. This discovery provides a method to release host genes hijacked by pathogens, opening new avenues for combating infectious diseases [123].

It was found by Zheng et al. [152] that the inhibitory impact of circYthdc2 against the antiviral immunity was primarily from its encoded Ythdc‐170aa. Overexpression of Ythdc2‐170aa was shown to promote the replication of Siniperca chuatsi virus (SCRV), a type of RNA virus. They applied a dual‐luciferase reporter vector system to demonstrate that this polypeptide primarily affected the activity of STING, thereby mediating the activity of antiviral immune‐related reporter genes such as type I IFN (IFN1), NF‐κB, IRF3, and ISRE. This discovery revealed the important role of Ythdc‐170aa in the antiviral immune response [152].

According to the findings reported by Wang and colleagues [205], during the infection process of the SCRV, in teleost fish, circMORC3 was observed to exhibit a different expression trend compared with its parental gene *MORC3*. In the early stages of infection, *MORC3* was found to respond positively, whereas circMORC3 shown no visible change. In the later stages of infection, the expression of *MORC3* recovered, but the levels of circMORC3 increased. Additionally, it was identified that circMORC3 encoded a polypeptide with antiviral activity, MORC3‐84aa. MORC3‐84aa was reported to effectively inhibit the TRIF‐mediated IFN1 and NF‐κB pathway activities by promoting the autophagy degradation of TRIF. This process was found to highly reduce the levels of antiviral cytokines, including inflammatory cytokines, IFNs and IFN‐stimulating genes, modulating the inherent immune activities against SCRV infection [205]. This study was noted to provide insights into the function of circRNA‐encoded polypeptides in antiviral innate immunity and offered new strategies for disease prevention and control.

**FIGURE 4:**
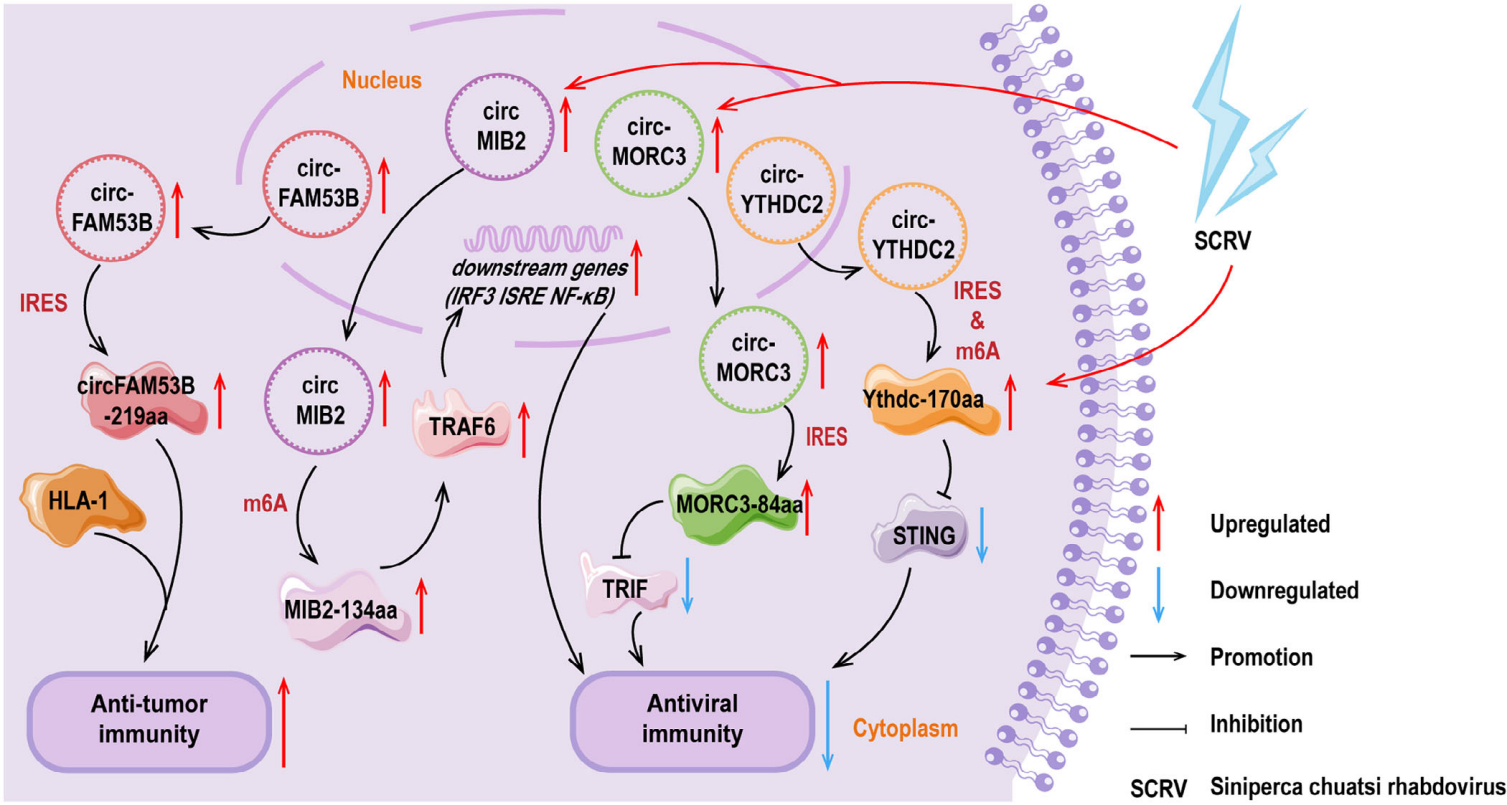
The schematic diagram illustrates that polypeptides encoded by circRNAs are implicated in the regulation of the immunity with disease. CircRNAs are represented by ring structures, and the polygon of the same color connected to circRNAs by an arrow represents the circRNA‐encoded polypeptides. Other polygons represent proteins, and the double helix shape represents DNA. Facilitation and inhibition are shown as arc‐shaped arrows, and upregulation and downregulation are shown as straight arrows. Lightning‐like shapes outside the phospholipid bilayer indicate exogenous stimuli. HLA‐1, major histocompatibility complex, class I; IRF3, interferon regulatory factor 3; ISRE, interferon‐stimulated response element; STING, stimulator of interferon genes; TRAF6, TNF receptor‐associated factor 6; TRIF, TIR‐domain containing adaptor‐inducing IFN‐β.

The details for the preceded points are rendered in Figure 4.

## 6 Therapeutic Applications Practice and Challenges of CircRNA‐Encoded Polypeptides

At present, therapeutic drugs based on circRNA‐encoded polypeptides are in the basic research stage, and a lot of research is still needed before clinical trials.

### 6.1 The Basic Exploration Stage before Clinical Application of CircRNA‐Encoded Polypeptides

After uncovering that circ‐EGFR‐encoded rtEGFRs promoted the development of GBM, Liu et al. [119] conducted a clinical animal trial of targeted knockdown of rtEGFR combined with nimotuzumab. The combination assay showed that nimotuzumab conferred a survival benefit in overall survival of mice when combined with the knockdown of rtEGFR [119]. Furthermore, Song et al. [133] found that circZKSaa enhanced the antitumor effect of sorafenib on HCC cells in vitro. It was found that the overexpression of circZKSaa further promoted HCC cell apoptosis after sorafenib treatment [133]. These results suggest that circZKSaa is expected to become a new peptide‐based therapeutic drug.

Zheng and colleagues [123] also revalidated the biological performance of MIB2‐134aa in zebrafish. The study found that circMIB2 successfully appeared in the liver, spleen, and kidney cells of zebrafish and encoded MIB2‐134aa. Additionally, the V. anguillarum infection assay found that zebrafish injected with Flag–circMIB2 had a stronger resistance to the colonization of Vibrio anguillarum. However, the organs of zebrafish injected with Flag–circMIB2–ATG–mut showed clear organs damage, and the bacterial infection was severe. These results collectively indicated that the MIB2‐134aa therapy enhanced the innate immune response in zebrafish, laying the foundation for the expansion of MIB2‐134aa as an exogenous polypeptide therapy [123]. These results exhibit the substantial potential of circRNA‐encoded polypeptides as the peptide drug in clinical therapy.

Pan et al. [206] found that high levels of hsa_circ_0007159 (circATG4B) circATG4B were presented in exosomes derived from drug‐resistant CRC cells and transmitted to nonresistant cells. After its translocation to the cytoplasm, it encoded circATG4B‐222aa, which acted as the key contributor to the oxaliplatin resistance of CRC. Specifically, circATG4B‐222aa functioned as a decoy for autophagy related 4B cysteine peptidase (ATG4B) protein and competed with transmemberane Emp24‐like trafficking protein 10 (TMED10), inducing the autophagy within recipient cells and reducing the pharmacological sensitivity of CRC cells to oxaliplatin [206]. This interventional treating targeting polypeptides encoded by circRNAs may provide a new perspective for improving or preventing chemoresistance of cancers.

TRIM1‐269aa, encoded by circTRIM1, was encapsulated into exosomes for intercellular transfer. Li et al. [207] found that TRIM1‐269aa was involved in mediating TNBC metastasis and chemotherapy resistance. It was found to enhance the mutual effect between MARCKS and calmodulin, facilitating calmodulin‐reliant translocation of MARCKS and activating the PI3K/AKT/mTOR pathway. This interaction promoted chemoresistance and metastasis in TNBC cells and tissues [207]. Similarly, there is promise for the development of peptide drugs targeting TRIM1‐269aa. The details for the relevant points are presented in Table 3.

**TABLE 3:** Research progress of circRNA‐encoded polypeptides in the treatment of diseases.

CircRNA	Polypeptide	Related mechanism	Potential clinical application	References
circ‐EGFR	rtEGFR	Targeted rtEGFR knockdowns in combination with nimotuzumab extended overall survival in mice	Offer a new combination therapy strategy for cancer treatment	[119]
circ‐ZKSCAN1	circZKSaa	Enhanced the antitumor effect of sorafenib on hepatocellular carcinoma cells and promote the apoptosis of hepatocellular carcinoma cells	Expect to be developed as a novel peptide anticancer drug	[133]
circMIB2	MIB2‐134aa	Enhanced the fish's resistance to bacterial infections	Offer the basis for developing exogenous polypeptide therapy	[123]
circATG4B	circATG4B‐222aa	Mediated colorectal cancer drug resistance and affects drug sensitivity through autophagy pathway	Offer a new idea for the intervention therapy of chemotherapy‐resistant tumors based on peptide drugs	[206]
circTRIM1	TRIM1‐269aa	Mediated TNBC metastasis and chemotherapy resistance	Offer the basis for the development of peptide drugs targeting TRIM1‐269aa	[207]

### 6.2 Risks and Considerations in the Application of CircRNA‐Encoded Polypeptides

CircRNA‐encoded polypeptides are natural peptides and are expected to become a new frontier of peptide drugs, enriching the clinical selectivity of peptide‐based therapeutics. However, this process is accompanied by some potential risks and side effects.

First, the artificial introduction of such polypeptides may be recognized by the immune system as foreign substances, causing an immune response that interferes with the therapeutic effect [208, 209]. Second, the polypeptides encoded by circRNAs may also have nonspecific binding with a variety of molecules in the body, interfering with normal physiological activities and causing side effects. In addition, as a peptide drug for clinical application, the drug dose of circRNA‐encoded polypeptides is a problem worth considering [210]. Excessive dosing may lead to toxic effects, whereas too low dosing does not guarantee therapeutic efficacy. Furthermore, the resistance of individual to chemotherapy drugs is common. Whether long‐term use of circRNA‐encoded polypeptides as therapeutic drugs will also develop disease resistance is worth further investigation [211, 212]. Moreover, researchers could leverage the unique properties of polypeptides to design effective and accurate drug delivery systems for diseases, while enhancing the bioavailability and the impact of treating with exogenous drugs by mimicking natural secretion pathways. For instance, TRIM1‐269aa was packaged into exosomes for intercellular transport and communication [207]. CircCCDC7‐180aa and circZKSaa were also detected in culture medium [133, 213]. Notably, circATG4B was presented in exosomes and encoded circATG4B‐222aa after delivery to the cytoplasm of recipient cells [206]. Finally, the utility and stability of these polypeptides as the peptide drugs are also an issue that should be considered by researchers and requires more comprehensive validation by researchers in clinical applications. In summary, it is even more matter for researchers to concern the transition from basic research to clinical applications, from disease detections to therapeutic strategies.

## 7 Conclusion and Perspectives

The market of peptide drugs is poised for stable and rapid growth, driven by the ongoing development of new technologies and the expansion of research fields [214, 215]. Basic research and clinical trials have shown the pivotal roles of peptide drugs in metabolic remodeling and immune response within the realm of diseases [216, 217]. They regulated the activity of relevant metabolic pathways and the level of response of the body's immune system.

Metabolic abnormalities refer to the disturbances in metabolism of glucose, lipids, amino acids (key amino acids like arginine, lysine, glutamine), and nucleotides [28, 216, 218]. Peptide drugs have been shown to balance energy metabolism and restore redox state by regulating the metabolic activity or mode of substrate molecules in the metabolic process [219]. Under normoxic conditions, cell thoroughly oxidize glucose molecules where oxygen enters into this oxidative mechanism as the final electron acceptor in the respiratory chain [220]. However, under hypoxic setting, cells shift to glycolysis to cope with the expanded energy demand, where the catabolism of glucose stops at lactate [51].

Lipids perform a dual role. They act as both cellular energy stores that feed energy metabolism and as components of the cell membrane, coordinating intercellular communication [221, 222]. Reprogramming of lipid metabolism is another metabolic adaptation mechanism distinguished by elevated uptake of foreign lipids, de novo lipogenesis, β‐oxidation of fatty acids, and storage of lipid droplet [223]. Peptide‐based therapeutics or inhibitors maintain the homeostasis of lipid content and lipid oxidation through these three pathways.

Amino acid metabolism is a normal metabolic activity of individual to maintain normal life activities, and orderly amino acid metabolism is necessary to sustain healthy metabolism [224, 225]. Abnormal glutamine metabolism is terminated by the inhibitors, exerting a beneficial effect. In addition, Wang et al. [226] found that another GLS inhibitor, BPTES, showed effective and specific inhibition of NLRP1b inflammasome activation in macrophages. Essentially, bis‐2‐(5‐phenylacetamido‐1,3,4‐thiadiazol‐2‐yl)ethyl sulfide (BPTES) offset the fate of NLRP1b proteasome degradation and induced subsequent caspase‐1 action [226]. These findings suggest that peptide drugs also cope with abnormal amino acid metabolism.

Peptide drugs exhibit precise and efficient mechanisms of action due to their high target specificity. This specificity not only reduces the risk of damage to healthy cells, but also highly reduces the occurrence of side effects. Studies have demonstrated that peptide drugs possess diverse antitumor mechanisms. They exerted antitumor activity by promoting tumor cell apoptosis [227], inhibiting tumor neovascularization [228], and activating antitumor immune response [229]. Moreover, the metabolites of polypeptide drugs are mainly amino acids, which are highly compatible with human metabolic processes and have good biocompatibility. This character significantly reduces the risk of immune response and toxicity in clinical applications. Additionally, peptide drugs also serve as diagnostic tools. Radiolabeled GX1 peptide has been shown to be useful for bioimaging of tumor angiogenesis [230]. However, several challenges remain in the development and application of peptide drugs. First, they exhibit poor stability in vivo and easily degraded by enzymes. Moreover, efficient delivery to target cells and avoidance of premature degradation in normal tissues still need to be explored. Additionally, the complex synthesis and purification processes result in high production costs, which limited their large‐scale application. Future research should focus on overcoming these limitations. Extensive research is currently being conducted on delivery strategies for peptide drugs. For instance, nano‐delivery systems, including liposomes and polymer nanoparticles, have been shown to enhance the stability and targeting capabilities of peptides [85]. Additionally, the chemical modification strategy of PEGylation enhances the half‐life of the peptide drugs and their circulation time in vivo. On the one hand, PEGylation increases the molecular weight of the peptide, slowing down its degradation rate in vivo. On the other hand, PEGylation forms a hydration layer to enhance its stability in the physiological environment. Meanwhile, with the deepening of research, novel disease targets have been gradually revealed, such as circRNA‐encoded polypeptides.

The current research results of circRNA‐encoded polypeptides are mainly concentrated in the field of cancer, where these polypeptides possess promotion effects or inhibitory effects on diseases [39]. Notably, the existing achievements of polypeptides encoded by circRNAs represent only the tip of the iceberg in this emerging field. In addition, van Heesch et al. [231] identified 169 unknown peptides translated from lncRNAs and 40 from circRNAs in human heart tissue, and the expression of these novel peptides can be extended to renal and hepatic tissues, indicating that their translation is a common event. Therefore, both the development of new peptide drugs targeting polypeptides and the design of therapeutic drugs based on polypeptides have broad research prospects and great clinical application value. Notable, the sequences of polypeptide encoded by circRNAs are largely consistent with those of their linear counterparts, with the exception of specific amino acid sequences that form minor variations at the cyclization site [112, 207]. Therefore, it is reasonable to synthesize peptide drugs targeting these specific amino acid sites in vitro.

In this review, we systematically summarized the underlying regulatory networks of peptide drugs involved in the regulation of metabolism and disease‐associated immunity activities in the individual. Peptide drugs have a wide range of applications and are common clinical drugs, but there is still room for optimization. Therefore, we outline the scheme of optimization of peptide drugs. At present, drug development based on circRNA‐encoded polypeptides has not been systematically conducted, but the existing molecular mechanisms have shown that circRNA‐encoded polypeptides have broad clinical application prospects. Therefore, we summarize the relevant encoding mechanisms of circRNAs, which are the fundamental principles that drive circRNAs to perform translation functions. In addition, based on the latest research findings, this review summarizes the specific mechanisms by which circRNA‐encoded polypeptides function in glucose and lipid metabolism, as well as in the metabolism of essential amino acids. In order to broaden our current understanding of circRNA‐encoded polypeptides. Moreover, we also summarize the initial application of circRNA‐encoded polypeptides as therapeutic methods in clinical animal experiments and sort out the possible problems in the development and application process. In order to provide certain reference value for the development of novel peptide drugs.

All in all, the refinement of the networks of peptide drugs, and the deepening of their roles in the mechanism of human diseases will provide new safeguard for human health.

## Author Contributions

Y.Q.H., and Y.K.Z. drafted the manuscript; Y.K.Z., and H.L. created the tables; Y.Q.H. drew the figures; Y.M.W. offered funding and performed writing—review and editing; Z.L.M. conceptualized the manuscript, conducted supervision, and writing—review and editing. All authors have read and approved the final manuscript.

## Conflicts of Interest

The authors declare no conflicts of interest.

## Data Availability

The data that support the findings of this study are available from the corresponding author upon reasonable request.
